# Breast Feeding Experiences of NHS Staff Returning to Work From Maternity Leave: A National Study

**DOI:** 10.1192/bjo.2022.199

**Published:** 2022-06-20

**Authors:** Hollie Hearfield, Jennie Collier, Fauzia Paize

**Affiliations:** 1 Ancora House, Chester, United Kingdom; 2 Wirral CAMHS, Wirral, United Kingdom; 3 Liverpool Women's Hospital, Liverpool, United Kingdom

## Abstract

**Aims:**

Anecdotally, NHS staff feel unsupported in breastfeeding when returning to work from maternity leave. The NHS provides clear guidance to employers about provisions required for breastfeeding employees (clean lockable room, adequate time, clean fridge). We aimed to establish if these provisions were provided for NHS staff, and to further explore the difficulties reported.

**Methods:**

We conducted a pilot study of NHS doctors, exploring their experiences of feeding on returning to work. The results highlighted difficulties for many of the 519 cases. We extended the study to encapsulate the experiences of all NHS professionals.

The survey was distributed via various professional social media accounts.

**Results:**

We received 1201 responses.

- 79% of women were breastfeeding when they returned to work. 59% wished to continue on return.
- 78% of women were unaware of the local breastfeeding policy. Of those that were, only 7% were informed of the policy by their employer.
- 90% of women were unaware that they needed to inform their employer of their intention to breastfeed.
- Only 6% of women had a breastfeeding risk assessment on their return to work.
- Basic requirements were not consistently met (50% did not have access to a lockable room, 51% to a fridge, 69% to adequate time).
- 55% were interrupted whilst expressing.
- 23% of women expressed in changing rooms; 32% in toilets; 25% in their cars; 15% in cupboards.
- 88% of women did not have their duties adapted. 91% regularly held the bleep whilst expressing.
- 52% of women reported embarrassment and humiliation at work. 60% reported stress directly due to their difficulties expressing, with a further 15% experiencing mental health problems. 10% of women felt their experiences negatively affected their bond with their child.

**Conclusion:**

Only 1% of UK mothers continue to breastfeed at six months. There is a huge NHS drive to improve this statistic. 76.7% of NHS staff are women. These women are also NHS patients. NHS breastfeeding guidelines are not being consistently followed within the organisation. There is a direct impact on mother and child, and on patient care.

We must support our NHS family, create a positive breastfeeding culture, and lead UK change.

“I was ridiculed… it set me apart from my colleagues.”

“Resigning was my only option.”

“Subject to eye rolls and whispers… rude, unsupported and unkind.”

“He unlocked the door and walked in while I shouted “stop”.”

